# Comparative Analysis of the Effects of Retirement on Health Status of Older Adulthood

**DOI:** 10.3390/ijerph19169957

**Published:** 2022-08-12

**Authors:** Liang Fang, Ruiyao Shi

**Affiliations:** School of Economics and Management, Beijing Forestry University, Beijing 100083, China

**Keywords:** health, retirement, discontinuity design, SAH, individual’s internal and external environment

## Abstract

This study examines the causal effect of retirement on the health of middle-aged and older adults in China. We used the mandatory retirement age in China as an instrument variable with a fuzzy discontinuity design. This served to examine the exogenous impact on retirement behavior. Two regression analyses were used, each with the dependent variable as self-assessed health status (SAH) and depression levels, respectively. Changes in an individual’s internal and external environment after retirement were associated with an increase in SAH and a decrease in depression. Highly educated men are 93.5% more likely to improve their health. Women are 0.26% less likely to be depressed. People with higher education tend to reduce their vigorous activities and increase walking for over 10 minutes after retirement. This study may promote research on aging and the health status of the Chinese population. It may provide a scientific basis for formulating, revising, and improving social security policies in China.

## 1. Introduction

For individuals who usually work in modern society, retirement marks the transition of social roles, social status, economic status, and living environments. It may impact the elderly on psychological and physiological levels. There is a long history of global research on how retirement impacts people’s health status. Yet, contradictions documented in existing global research on how retirement affects health validate the significance of continued research to better understand short-term and long-term economic, sociological, and public health ramifications [1,2,3,4,5]. In Japan, research suggested that the psychological benefits of retirement did not persist for extended periods after retirement [4]. In Hong Kong, research reaffirmed the need for “policymakers and service providers to improve public education to raise future retirees’ awareness of the importance of retirement planning and promote retirement planning activities, especially social life planning” [3] to promote lasting satisfaction. From a social science and shared knowledge perspective, retirement frees one from time-consuming and stressful work. This freedom is beneficial to one’s health. Conversely, retirement may inevitably simplify daily social relationships and change one’s pace of life. The induced loneliness may reduce the sense of self-efficacy, which may be harmful to one’s health. Therefore, it is important to explore the impact of retirement on the elderly in China’s health status and its extent.

Unlike some western developed countries, China has a mandatory retirement age policy. The age set by the retirement policy largely determines when the labor force will permanently leave the labor market. In this context, the impact of retirement on the physical and mental health of middle-aged and elderly people in China is important. The health changes of people who voluntarily apply for early retirement and internal retirement before and after retirement are insignificant. Conversely, the changes in people forced to leave the labor market due to policy regulations are significant. Therefore, it is important to determine the impact of retirement on their health for policy and medical reference value purposes. This is not only for evaluating people’s health status post-retirement but also for the potential retirement age adjustment.

Econometric models have been used in most related studies to conduct empirical research on authoritative databases. Regarding the choice of identification methods, most earlier studies [6] used ordinary least squares in a regression analysis on health indicators and retirement. Instrumental variable (IV) and regression discontinuity (RD) estimators are commonly applied to avoid the underlying simultaneous causality problem. This serves to control for the endogeneity of the impact of retirement on health status. This helps solve other challenges [7,8,9]. Some scholars found that retirement decreased men’s health but had no significant effect on women in China [10]. However, Ekerdt D et al. [11] have shown that retirement significantly improves the health status of men with reduced job stress and roles post-retirement. A number of European academics [12] examined the Survey of Health, Aging and Retirement in Europe (SHARE) dataset in Europe. They found that retirement significantly improved comprehensive health indicators. Deng and He [13] have found that retirement had no significant effect on individuals’ physical health. However, it had a significant positive effect on mental health.

Possible rationales for the different results may be the sample heterogeneity and various channel mechanisms. This is with the exception of the differences in econometric methods and sampled regions. Scholars have studied the tools and channels of action from changes in health behavior. These include exercise, social activities, smoking, drinking, and sleeping schedules after retirement. Although this research mainly focused on developed countries, discussions on China have increased in recent years. For example, in the study of Chinese cities, gender differences in retirement age were found to be the underlying mechanism of heterogeneity [7]. Retirement had a more significant positive effect on cognitive levels and mental health for male blue-collar workers. They would engage in more social activities and travel when they had more leisure time after retirement. Therefore, Su and Li [8] found that a significant increase in social activity opportunities after retirement increased the male employees’ health status. However, the decrease in chronic diseases in women could not be explained. Zheng and Wang [14] noted that retirement reduced men’s health. This was due to the significant reduction in health behaviors, social activities, and cognitive abilities after their retirement. Zou Hong et al. [15] showed that for the elderly who smoked and drank, there was a significant decrease in their tobacco and alcohol consumption after retirement. Conversely, Feng and Li [9] found that retirement significantly increased men’s weight and BMI index with lower education levels. This is possible because they were more likely to drink alcohol after retirement and do vigorous exercise less frequently.

In a study on the impact of delayed retirement on the elderly’s health, Huang and Yu [16] found that retiring without resting could significantly improve the health of the young-old. No significant effect on the oldest-old in overall health status was determined. However, a significant adverse effect on their cognitive abilities was found. From a labor participation perspective, Wan et al. [17] found that labor participation behaviors improved the retired elderly’s self-comment health and daily living abilities. They also reduced their chronic diseases. Studies on the health effects of spousal retirement showed that it significantly improved men’s self-assessed health status (SAH). It had a significant positive spillover effect on both gender’s mental health. The main reason was that after the spouse’s retirement, the husband would receive more care. The frequency of exercise for both spouses increased [18].

There are diverse opinions on the causal effects of retirement on health. China’s research on health-dependent variables based on tests is still insufficient. The analysis of mental health and depression is still primitive. The relevant mechanism and channel analyses are elementary, and the data are not updated in a timely manner. Considering China Health and Retirement Longitudinal Study (CHARLS)’s updated frequency, we can supplement its latest data in 2018. This was made known to the public at the end of 2020 [19]. Hence, this study intends to add depression as a dependent variable. It also uses the CHARLS’ data from 2018 to discuss the mechanism of retirement on health. This occurs in the context of China’s delayed retirement policy. The remaining paper is organized as follows:

- The institutional background and theoretical hypothesis are illustrated in Section 2.
- Descriptions of the data source, data processing, and variable settings are provided in Section 3.
- An empirical analysis of the impact of retirement on health is presented in Section 4. This includes the model’s construction of the impact of retirement on health, and the heterogeneity of the role of retirement. It also consists of the analysis of potential mechanisms and channels.
- The conclusions and future research recommendations are discussed in Section 5.

## 2. Institutional Background and Theoretical Hypothesis

The United States implemented a flexible retirement age policy. Upon retirement, employees become financially dependent on the government and stop working. Concurrently, the flexible retirement age policy has a flexible pension age. It also offers a re-employment policy that can be independently chosen. However, this design mainly considers economic benefits [20]. However, in China, most employees have to leave their employment positions after completing the retirement procedures. Although a few high-tech experts remain in their posts due to re-employment, most people experience difficulty finding another job. Therefore, discontinuity in China’s retirement rate is highly probable. In China, the retirement rate is not a zero to one jump under the mandatory retirement age but a significant increase in the possibility. This is a typical fuzzy discontinuity regression in econometrics [21].

Under the local average treatment effect (LATE) framework of econometrics, the population is divided into four categories. These are based on the population’s response to the IV:

- Compliers refer to people who do not retire before 60 years but retire after 60 years;
- Defiers refer to people who retire before 60 years but do not retire after 60 years;
- Always-takers refer to people who will retire regardless of whether they have reached 60 years of age;
- Never-takers refer to people who do not quit, irrespective of whether they have reached 60 years.

There is a possibility that the “compliers” identified by the mandatory retirement age as an IV may only be some of these individuals. However, they are not the population we are concerned with. In China, compliers are our main concern. This is because the study examines the causality of retirement to health status. The aim is to offer theoretical guidance to adjust the national retirement policy and improve the elderly retirees’ welfare. Under positive monotonicity, there will be no opt-out situation when the qualification is granted. Therefore, we are justified in asserting that there are no defiers in the population [22]. In most cases, such a monotonicity assumption is reasonable, as supported by Lei Xiaoyan et al. [10]. Despite the reluctant simplification in China, the “compliers” group is practically essential to our understanding of the overall population.

Regarding the RD analysis, Lei Xiaoyan et al. selected SAH. They set 50, 55, 60, and similar neighborhoods, as the observation intervals. This is in accordance with the existing Chinese retirement policy. The results showed that the health level showed non-smooth changes at the retirement age nodes. This study is based on a sample with a significant difference before and after the mandatory retirement age [10]. The RD design’s premise is that the variables are smoothly affected by the natural effects over time. However, a sudden and statistically significant change, is formed at a particular time. This may be a result of the exogenous factors. We will evaluate the retirement dummy variable and add IVs to the two-stage regression. This serves to obtain the effect of retirement on health. It changes into various variables after retirement.

## 3. Data Procession and Variable Settings

The study’s data were obtained from the baseline national wave data of CHARLS from Peking University. The baseline national wave of CHARLS was conducted in 2011. It includes households and individuals in nearly 150 counties/districts and 450 villages or resident committees. The individuals were and will be followed up on every two years. The questionnaire contained the following modules: demographics, family structure/transfer, health status and functioning, biomarkers, retirement and pension, income and consumption, etc. The high-quality micro-data it provides will greatly promote interdisciplinary research on aging and health. It will contribute to the formulation, revision, and improvement of social security policies in China.

The four-year panel data are individually completed each year. It is complex to show the sample selection process directly. Therefore, by using the 2015 data as an example, we performed the following data processing series:

1. After merging and constructing the necessary variables, we removed samples with BMIs that were more significant than 35 (281 observations were deleted). This was due to the possibility of sample recording errors and the limitations of human physiological characteristics;
2. Considering this study’s purpose, we deleted samples younger than 45 years and older than 70 years (3186 observations were deleted);
3. There are 122 observations with missing SAH. After dropping these missing values, we excluded those who had only engaged in farm work or were never in the labor market (4238 observations are deleted). This was since there is no definition of retirement for people with a rural residence. This includes people who only did farm work or had never worked in the labor market. We also excluded those whose working status will not change after their retirement. People with rural residences were also removed (4595 samples were deleted);
4. We removed the observations where the data of the household food and total household expenditures were missing or had extreme outliers (We removed 144 comments);
5. We obtained 2079 observations in 2015.

By using a method similar to the processing of the 2015 data, we obtained four-year panel data. This had a total of 7608 observations after revising the questionnaire for each year. We set the bandwidth to [−5, 5]. This was based on the RD design’s premise and the local experimental design’s interval requirements. Therefore, all the observations were within five years of the retirement age. Finally, 3407 observations were left. Table A1 shows the dictionary of the corresponding variables. It aims to explain each variable’s meaning. Table A2 presents the related variables’ descriptive statistics. From these statistics, among the 3407 observations, the average age was 55.4 years old. The percentage of people who thought they were healthy was 46.9%. The average depression index was 20.29. Approximately 41.9% of the observations were drinkers. Of the observations, 92.3% were married or living with their spouses. The dictionary of the corresponding variables and descriptive statistics are are shown in Table A1 and Table A2 in the Appendix B.

### 3.1. Health

In this study, SAH and depression are the health indices. In the literature on the causal effects of retirement on health, many indicators have been used to reflect health status. These include SAH [8,10] and BMI [9], cognitive abilities [7,23], comprehensive health indicators [12], and so on. The main reason for refusing BMI is that a healthy BMI is a range. This makes it difficult to explain whether it is healthy. Conversely, SAH includes both physical and psychological dimensions. This reflects the individual’s cognition and evaluation of the existing diseases. It also indicates those undiagnosed diseases that present symptoms. Even the harshest critic could not omit its analysis [10]. Therefore, we chose the SAH status as the primary dependent variable in this study. The value of SAH in the CHARLS questionnaire ranges from one to six. This represents “excellent, very good, good, fair, poor, and very poor”. The higher the value, the worse the health level. By using binary variables in the previous literature [8,10], we set one-three points to one to indicate good health. We set four-six points to 0 for being unhealthy. Therefore, the retirement variable’s coefficient represents a change in the SAH’s status after retirement.

Furthermore, since their physical health may not suddenly worsen upon retirement, its impact on health may be gradual. This is especially on the spiritual and psychological levels. We then added the depression scale to reflect their mental health. The Center for Epidemiological Survey-Depression (CES-D) can intuitively measure people’s mental health and reflect personal subjective feelings. CHARLS also provides CES-D data. We generated the depression variable based on the data from the cognitive and depression modules. After screening out the questions with no definite answer, we reversed the inverse questions. This was conducted according to the simple depression scale measurement method. We then calculated the total. For the imputation of the missing values, we first averaged the items’ scores that were not missing for the same individual. We then multiplied this by 10. Finally, we multiplied them by 1.25 to place the value in the interval of 12.5 to 50. The value is proportionate to the degree of depression and inversely proportional to mental health.

### 3.2. Health Behaviors

We had set drinking as a dummy variable: one means as long as they continue to drink and otherwise 0. Regarding exercise, based on the “Vigorous activities” selected by Feng and Li, this article further supplements “Moderate activities”. These are moderate-intensity physical activities [9]. We also added “Socialist”, namely “Social activities”, and “Walking more 10 min”, as dummy variables of healthy behavior. This served to explore underlying mechanisms and channels.

### 3.3. Independent Variables

Regarding the control variables, we included: retirement, age, gender, marital status, residence, consumption level, education level, household number, and number of children. Regarding the age details, we set the variable “Age” as the distance between the actual age minus the mandatory retirement age (60 years for men and 50 years for women). This was due to the differences in the retirement age and traditional household responsibilities between the sexes.

Considering the major impact of the education level on people’s living standards, education is a good way of grouping. People with higher education have a more comprehensive knowledge and a more robust internal drive for healthy behaviors. They also have more self-regulation abilities after retirement [10]. We classified “illiterate”, “did not finish primary school”, “elementary school”, and “middle school” as lower education. “High school” and above were classified as higher education. We assumed that each interviewee’s education level did not change over time. This simplified our missing value filling procedure. This was applied to all middle-aged and older adult interviewees older than 45 years. We completed the missing values according to their educational level in different years.

No variables were used to measure the level of comprehensive personal income in the CHARLS questionnaire. The length of the income-related questions and the interviewees’ possible “underreporting” tendency are important factors. They may therefore be measurement errors and resultant statistical difficulties. Consequently, we constructed the variables of household consumption and the total annual expenditure of the family. This is used as a comprehensive reflection of family income. We specifically multiplied the weekly food expenditure by 52 weeks. We then added the total monthly spending, multiplied by 12 months. The total annual spending multiplied by one was conducted to obtain the entire household’s consumption expenditure in the past year. They cannot grow their food in rural areas because the interviewees we discuss are all located in cities. Even if there is a small vegetable garden in the town that grows vegetables, it is negligible. Other demographic variables in this study are defined as follows: marital status is divided into married (living with a spouse) and the other. The questionnaire can directly obtain information. These include gender, residence, sleeping time, number of family members, and number of children in the household.

## 4. Empirical Analysis of the Impact of Retirement on Health

### 4.1. Endogeneity

Before exploring the causal impact of retirement on health, we first discuss the endogeneity of the regression model. Previous literature emphasized three endogenous problems: omitted variable bias, simultaneous causality, and measurement error. We will address these individually.

When exploring the impact of retirement on health, the omitted variable bias comes from variables related to the independent variable (retirement). It also comes from the dependent variable (health). These variables are often unobservable and cannot be included in the regression model. These include genetic factors and life expectancy. We use panel data with individual fixed effects to address omitted variables that do not change with time. Currently, the deviation and heterogeneity of omitted variables that do not change with time can be controlled.

There is also an apparent simultaneous causality problem: whether one’s health is good or not will affect the decision to retire. For example, if one’s health is good, they may want to work for a few more years and retire later. People with poor health may wish to retire earlier. This makes it difficult to identify causal effects. Therefore, we adopt the method of fuzzy discontinuity regression and select the mandatory retirement age. This has been commonly used in the literature as the Instrumental variable (IV) in performing a two-stage regression to solve the problem of simultaneous causality [7,9,10].

The measurement error problem has also been discussed in the literature. The focus of the debate is whether to choose an objective measurement index or a subjective evaluation index. There is often a classic measurement error (attenuation bias) in objective health indicators, such as height and weight. This is caused by the nature of CHARLS, as a follow-up survey. Different interviewers in different years may measure the size and weight of the same respondent using other standards. The primary dependent variable in this study was self-comment health. This is a subjective evaluation index. The measurement error of this type of index originates mainly from justification bias. McGarry mentioned that people might exaggerate the severity of their suboptimal health to justify their retirement decisions [24]. This study also adds a relatively objective depression scale index as an essential dependent variable. This serves to measure mental health to solve this problem. This test-based health evaluation method is relatively accurate. This reduces the possibility of justification deviation. It even reduces the deviation of the least-squares estimator.

### 4.2. The Designing of the Regression Discontinuity Regression

**Figure A1:**
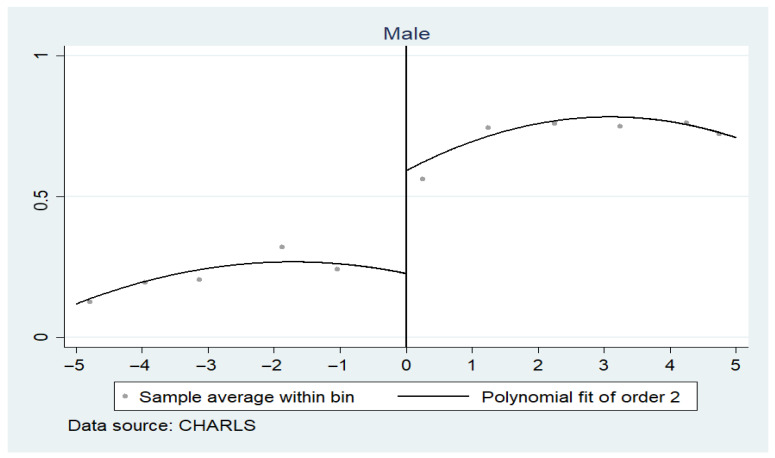
Discontinuity diagram of the average retirement rate of men by age.

**Figure A2:**
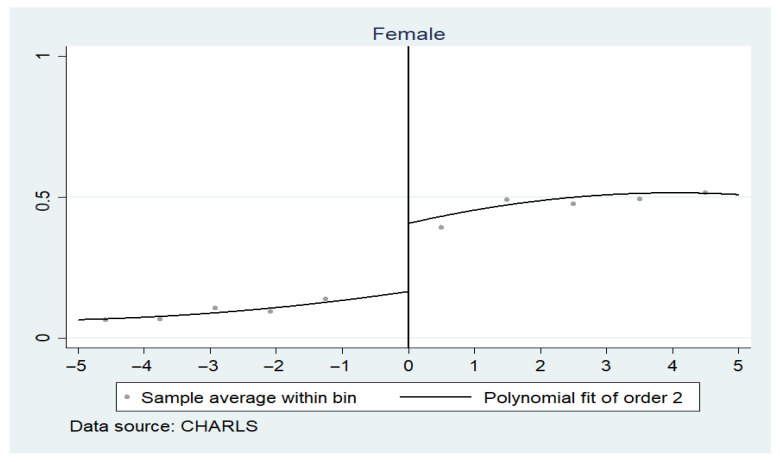
Discontinuity diagram of the average retirement rate of women by age.

We chose 60 years old as the discontinuity value for men’s retirement and 50 years old as the discontinuity value for women’s retirement. By using Stata14, we obtained Figure A1 and Figure A2 (See in the Appendix A). Figure A1 shows the average retirement rate of men of different ages. The X-axis represents the distance from the actual period to the mandatory retirement age. The y-axis represents the real retirement rate of men and women. The curve fitting results show that the actual retirement rate has risen from 20% to 55% before and after for men (60 years old). Figure A2, shows that the retirement rate increased from approximately 15% to 40% for women. The actual retirement behavior of men and women is consistent with their retirement ages. This establishes the completeness and rationality of our RD design.

### 4.3. Model of the Impact of Retirement on Health

Equation (1) represents the first stage of the regression. $R_{it}$ is the data in the questionnaire. It indicates whether person *i* has retired in year *t*. $E_{it}$ is a dummy variable, indicating whether an individual’s age exceeds the mandatory retirement age. If their age exceeds the institutional retirement age, it is equal to one. Otherwise, it is 0. According to the previous narrative, we set 60 years of age for men and 50 years of age for women as the mandatory age. The variable “Age” represents the distance from the real age to the eligibility retirement age. $X_{it}$ represents time-varying individual characteristic variables. This includes marital status and residence. $Z_{it}$ is a variable reflecting family characteristics (e.g., the largeness of a household). $\alpha_{i}$ is the individual fixed effect, and $u_{it}$ is the error term. Table A3 in the Appendix B shows the first-stage estimation’s results.

(1) $$R_{it}=\omega_{it}+\omega_{1}E_{it}+\beta_{1}Age+\rho X_{it}+\eta Z_{it}+\alpha_{i}+u_{it}$$

We further explain the linear probability model’s rationality. The Heckman two-step method is an effective method to address sample selection bias. Regardless of the previous literature or the nature of the CHARLS, this endogeneity problem is not severe. We treat the SAH as a dummy variable. We then use the linear probability model directly, as the current ordinary least squares estimator is still consistent. Furthermore, the coefficient can be directly explained, regarding how much change in the probability of y=1 will result when changing the value of *x*. This is when other factors remain unchanged. However, if nonlinear models, such as probit and logit are used, the coefficients themselves are meaningless. The marginal effects must then be calculated. Currently, the estimator is a maximum likelihood estimator. There are no analytical solutions. This will inconvenience the calculation and estimation. Therefore, in this study, we chose the linear probability model.

Table A3 (See in the Appendix B) presents the first-stage regression’s results. Robust clustering standard errors at the individual level are reported in parentheses. The first-stage regression is essentially a matching evaluation on an “individual’s actual retirement behavior” and “whether it reaches the eligibility retirement age”. The F-statistic value of the first-stage regression is greater than 10. This indicates that the IV is influential. As the actual age reaches the mandatory retirement age, men’s retirement rate increases by 30%. Women’s retirement rate increases by 19.9%. Both are significant at the 1% significance level.

(2) $$Y_{it}=\omega_{it}+\lambda {\hat{R}}_{it}+\beta_{1}Age+\rho X_{it}+\eta Z_{it}+\alpha_{i}+\epsilon_{it}$$

Equation (2) shows the second-stage regression design: here, ${\hat{R}}_{it}$ refers to the predicted value of the first-stage regression. It also refers to the other variables that are similar to the one-stage regression. Table A4 (See in the Appendix B) shows that from a gender perspective, retirement has a positive effect on the SAH in both men and women. The men’s coefficient is 0.220. This is positively correlated. The women’s coefficient is 0.432. Both outcomes are not statistically significant). This shows that people’s health status will rise after retirement. However, the degree of the effect of retirement on health varies between men and women. When depression is used as the dependent variable, retirement reduced the degree of depression in men and women (by 0.186 and 0.26 points, respectively). However, these two effects are not significant.

### 4.4. Heterogeneity

Since the initial regression’s results are not significant, we speculate that there may be heterogeneity among different groups of people. Therefore, we further group the samples according to their education level. We then performed a regression analysis. Table A5 (See in the Appendix B) shows that the possibility of being a healthy man with lower education levels decreases by 32.1% after retirement. Conversely, men with higher education levels and all women were more likely to fit into the group after retirement. In particular, the likelihood of being a healthy man with higher education levels increases by 93.5% (at a significance level of 5%) after retirement.

The possibility of being healthy increases with age. The results are: 0.087 (under the significance level of 1%), 0.053 (under the significance level of 10%), 0.042, and 0.08 (under the significance level of 1%). Our explanation for this is that men with lower education levels may engage in more physical activities. They may also do less exercise and be involved in fewer social activities after retirement, than their higher education level counterparts. It takes a longer time for them to become familiar with the new life rhythm and environment. Due to relatively large psychological fluctuations, they may have a pessimistic attitude toward their health. However, men with higher education levels and all women are more likely to increase their leisure time. It is much easier for them to balance psychological changes and sustain their new sustenance in life. The specific mechanism and channel analysis will be elaborated on below.

### 4.5. Analysis of Underlying Mechanisms and Channels

The research above shows that retirement positively affects the SAH status and depression in men and women. However, we wish to examine what the underlying mechanism is. Therefore, channel analysis can be performed. Considering that the sample size of each category is small, when we group the sample by education and gender, it may affect the significance level. However, grouping by education level alone does not affect the existing significance. Table A6 (See in the Appendix B) shows that the probability of being healthy for highly educated people after retirement increases by 51.5%. Therefore, in the channel analysis, we only considered grouping based on the level of education.

We used vigorous activity, moderate activity, social act, drinking, and walking for more than 10 minutes, as dependent variables. This served to perform the same two-stage regression as per above. Table A7 (See in the Appendix B) shows that the probability of vigorous activity for people with higher education reduces by 30% (under a significant level of 10%). It also shows that the likelihood of walking increases by 25% after retirement. According to the theory of channel analysis, if the mechanism is from the channel variable when this variable is controlled, the significance level of λ (namely the coefficient of retirement on health) will change. The absolute value may decrease instead. Based on this, we controlled vigorous activity and walking for more than 10 minutes for people with higher education. This served to identify the changes in the impact of retirement on health.

Table A8 (See in the Appendix B) indicates that after adding the control variable vigorous activity, λ (the coefficient of the impact of retirement on self-comment health) drops by 4.3% (0.515 − 0.472 = 0.043). This is at a 10% significance level. However, the coefficient of vigorous activity is not significant. This indicates that this channel’s explanatory ability is general. After adding walking for more than 10 minutes, λ was reduced by 3.8% (under the 10% significance level). Concurrently, the coefficient of walking for more than 10 minutes was 0.156. This was significant at the 5% significance level. The results show that for people with higher education, for every percent increase in the probability of walking, the probability of being healthy increases by 0.156%. This shows that for highly educated people, the impact of retirement on their health is mainly determined by the increase in the probability of walking after retirement. It is also partly determined by a decrease in vigorous activity. This further validates the previous hypothesis: that people with high academic qualifications are more likely to adapt to retirement life with more leisure time. They may also consciously increase their walking time and reduce time spent on energy-consuming vigorous activities, to improve their health.

## 5. Conclusions

The study indicates that post-retirement, both genders’ physical health may improve. The degree of depression also decreased. However, this is a non-significant effect. From a heterogeneity analysis perspective, for people with higher education, the possibility of being healthy after retirement increases by 51.5% (at a significance level of 10%). This is especially for highly educated men, whose average effect is 93.5% (at a significance level of 5%). From a channel analysis perspective, highly educated people significantly reduced their frequency of vigorous activity. They also increased their probability of walking for more than 10 min. This is the main reason for improving the health of highly educated people.

However, the study has some limitations: We are concerned about retirement’s short-term average causal effect. Retirement’s impact on depression or health status may be delayed. This may be related to behavioral adjustments post retirement [7]. Retirement may have a “honeymoon period” for cognitive levels and depression. Relief from stressful work pressure may reduce some of retirement’s negative effects [23]. Retirement may suppress cognitive decline in the short-term. However, in the long-term, retirement may reduce cognition [7]. Apart from gender and education, there may also be other grouping methods that may significantly affect the results. These include health and financial status at retirement and the number of household members. We might consider leveraging our results to contribute to the broader global challenge of promoting “physical health and life satisfaction through increased retirement confidence” [3]. Finally, the choice of different bandwidths, different definitions of retirement, and retirement age may change the results’ significance. Thus, a relevant robustness analysis needs to be explored in the future.

## Data Availability

The study’s data were obtained from the baseline national wave data of CHARLS from Peking University at https://opendata.pku.edu.cn/dataverse/CHARLS (accessed on 10 July 2022).

## Abbreviations

The following abbreviations are used in this manuscript:

SAH	Self-assessed health status
IV	Instrumental variable
RD	Regression discontinuity
CES-D	The Center for Epidemiological Survey-Depression
CHARLS	China Health and Retirement Longitudinal Study

## Appendix B

**Table A1:** Dictionary of the Corresponding Variables.

Variable Name	Variable Meaning	Detailed Description	Range
Age real	Real age	Continuous variable	45∼65
Age	Age gap	Continuous variable	−5∼5
Rgender	Gender	Qualitative variable with 2 levels	1 = Male
			2 = Female
Marriage	Marriage	Binary variable	1 = Married or lived with spouse
			0 = otherwise
SAH	Self-assessed health	Binary variable	1 = Good
			0 = Bad
Depression	Degree of depression	Continuous variable	12.5∼50
Retirement	Retirement status	Binary variable	1 = Retired
			0 = Not retired
Edu level	Education level	Binary variable	1 = Higher education
			0 = Lower education
House hold num	House hold numbers	Discrete variable	−
Child in house	Children who live together	Discrete variable	−
Drinking	Vigorous activity	Binary variable	1 = Drink now
			0 = Not drink now
Vigorous activity	Moderate activity	Binary variable	−
Moderate activity	Walking more 10 min	Binary variable	−
Walking more 10 min	Log total expenditure	Binary variable	−
Log total expenditure	Socialact	Continuous variable	−
Socialact	Sleeptime	Binary variable	−
Sleeptime	Vigorous activity	Continuous variable	−

**Table A2:** Descriptive Statistics.

Variable	N	Mean	Std.	Min	Max
Marriage	3407	0.923	0.267	0	1
SAH	3407	0.469	0.499	0	1
Depression	3407	20.285	6.774	12.5	50
Retirement	3407	0.386	0.487	0	1
Edu level	3407	2.251	0.738	0	3
House hold num	3407	0.842	0.965	0	7
Child in house	3407	0.352	0.613	0	4
Drinking	3407	0.419	0.494	0	1
Vigorous activity	3407	1.826	0.298	1	2
Moderate activity	3407	1.581	0.434	1	2
Walking more 10 min	3407	1.393	0.429	1	2
Log total expenditure	3407	7.546	0.951	3	11.6
Socialact	3407	0.709	0.454	0	1
Sleeptime	3407	6.975	1.728	0	15

Note: The variables initially considered to be retained are health indicators, which are mainly divided into three dimensions: health variables, health behavior variables, and independent variables.

**Table A3:** First stage estimation results by gender and education of being retired.

	Male	Female	Lower Education	Higher Education
	(1)	(2)	(3)	(4)
e	0.300 ***	0.199 ***	0.247 ***	0.300 ***
	−0.059	−0.059	−0.053	−0.07
Age	0.003	0.007	0.004	−0.001
	−0.011	−0.01	−0.009	−0.012
Age2/100	−1.011 ***	−0.555 ***	−0.624 ***	−1.033 ***
	−0.187	−0.176	−0.164	−0.219
Marriage	−0.034	0.02	−0.03	0.047
	−0.102	−0.145	−0.126	−0.177
House hold num	−0.022	0.001	0.002	−0.031
	−0.014	−0.017	−0.015	−0.02
Edu level	0.042	−0.048		
	−0.052	−0.085		
Log total expenditure	0.005	−0.005	0	0.007
	−0.022	−0.02	−0.018	−0.027
Cons	0.348	0.38	0.332 *	0.302
	−0.222	−0.291	−0.178	−0.255
N	1677	1518	1922	1273
F	14.76	7.098	10.49	14.22

Note: (1) Standard errors are in parentheses. (2) * *p* < 0.1; *** *p* < 0.01. (3) The result is estimated by fixed effect model with panel data. (4) We also control marriage, household number, log total expenditure and individual fixed effect.

**Table A4:** Effect of retirement on SAH and depression by gender.

	Male SAH	Male Depression	Female SAH	Female Depression
	(1)	(2)	(3)	(4)
Retirement	0.22	−0.186	0.432	−0.26
	−0.208	−2.613	−0.359	−3.931
Age	0.067 ***	0.024	0.064 ***	0.209
	−0.012	−0.146	−0.014	−0.147
Age2/100	−0.211	−1.369	0.283	−3.454
	−0.256	−3.054	−0.264	−3.016
Marriage	0.288	−5.339 **	0.088	−2.544
	−0.193	−2.429	−0.16	−1.85
House hold num	0.046 **	−0.662 ***	0.007	0.115
	−0.02	−0.213	−0.024	−0.261
Edu level	0.035	0.435	0.096	−0.372
	−0.044	−0.93	−0.091	−1.224
Log total expenditure	0.043 *	−0.148	0.03	0.115
	−0.026	−0.299	−0.033	−0.269
Cons	−0.328	25.554 ***	−0.211	23.587 ***
	−0.281	−3.669	−0.391	−3.794
N	1677	1668	1518	1517

Note: (1) Standard errors are in parentheses. (2) * *p* < 0.1; ** *p* < 0.05; *** *p* < 0.01. (3) The result is estimated by fixed effect model with panel data. (4) We also control marriage, household number, log total expenditure and individual fixed effect.

**Table A5:** Effect of retirement on SAH by education level.

	Male SAH	Female SAH
	(1)	(2)
**Lower education**		
Retirement	−0.321	0.667
	−0.28	−0.527
Age	0.087 ***	0.053 **
	−0.014	−0.021
Age2/100	−0.589 *	0.298
	−0.32	−0.343
Marriage	0.197	0.077
	−0.207	−0.297
Householdnum	0.023	−0.01
	−0.02	− 0.04
Log total expenditure	0.060 *	0.089 *
	−0.035	−0.049
Cons	−0.082	−0.465
	−0.323	−0.433
N	1045	877
**Higher education**		
Retirement	0.935 **	0.135
	−0.414	−0.419
Age	0.042	0.080 ***
	−0.027	−0.017
Age2/100	0.671	0.088
	−0.541	−0.415
Marriage	0.673 **	0.296 *
	−0.269	−0.172
Householdnum	0.108 **	0
	−0.045	−0.033
Log total expenditure	0.027	−0.069
	−0.055	−0.046
Cons	−0.785	0.725 *
	−0.535	−0.377
N	632	641

Note: (1) Standard errors are in parentheses. (2) * *p* < 0.1; ** *p* < 0.05; *** *p* < 0.01. (3) The result is estimated by fixed effect model with panel data. (4) We also control marriage, household number, log total expenditure and individual fixed effect.

**Table A6:** Effect of retirement on SAH by education.

	Lower Education	Higher Education
	SAH	SAH
	(1)	(2)
Retirement	0.039	0.515 *
	−0.232	−0.279
Age	0.072 ***	0.060 ***
	−0.011	−0.015
Age2	−0.157	0.215
	−0.212	−0.338
Marriage	0.096	0.331 *
	−0.14	−0.176
Householdnum	0.02	0.039
	−0.018	−0.031
Log total expenditure	0.063 **	−0.021
	−0.025	−0.036
Cons	−0.178	0.18
	−0.222	−0.335
N	1922	1273

Note: (1) Standard errors are in parentheses. (2) * *p* < 0.1; ** *p* < 0.05; *** *p* < 0.01. (3) The result is estimated by fixed effect model with panel data. (4) We also control marriage, household number, log total expenditure and individual fixed effect.

**Table A7:** Effect of retirement on health behaviors by education.

	(1)	(3)	(5)	(7)	(9)
	Vigorous Activity	Moderate Activity	Walking More 10 min	Socialact	Drinking
**Lower education**					
Retirement	0.029 (0.132)	0.005 (0.210)	−0.219 (0.212)	−0.330 (0.233)	0.227 (0.198)
Age	−0.013 ** (0.007)	0.026 ** (0.010)	0.078 *** (0.011)	0.004 (0.011)	−0.007 (0.009)
Age2/100	0.087 (0.142)	−0.042 (0.195)	0.072 (0.196)	0.000 (0.219)	0.111 (0.182)
Marriage	0.026 (0.035)	0.077 (0.099)	0.107 (0.107)	−0.083 (0.171)	0.036 (0.068)
House hol dnum	−0.005 (0.008)	−0.007 (0.015)	0.010 (0.018)	−0.000612	−0.001 (0.013)
Log total expenditure	0.009 (0.011)	−0.011 (0.020)	0.013 (0.022)	0.043 ** (0.021)	−0.006 (0.021)
Cons	0.079 (0.099)	0.409 ** (0.184)	0.422 * (0.220)	0.557 ** (0.236)	0.307 (0.193)
N	1922	1922	1922	1922	1921
**Higher education**					
Retirement	−0.052073	0.008 (0.233)	0.249 (0.218)	−0.059 (0.203)	0.226 (0.200)
Age	0.008 (0.010)	0.029 ** (0.014)	0.060 *** (0.013)	0.004 (0.011)	−0.002 (0.011)
Age2/100	−0.435 ** (0.220)	−0.302 (0.292)	0.034 (0.263)	−0.102 (0.248)	0.250 (0.252)
Marriage	−0.144 (0.114)	0.016 (0.179)	−0.065 (0.146)	0.083 (0.183)	−0.030592
House hold num	0.001 (0.019)	0.011 (0.025)	0.008 (0.025)	−0.000792	0.014 (0.028)
Log total expenditure	0.004 (0.018)	0.058 ** (0.026)	0.060 ** (0.024)	0.029 (0.027)	0.020 (0.024)
Cons	0.429 ** (0.193)	0.013 (0.275)	0.166 (0.230)	0.558 ** (0.259)	0.393 * (0.219)
N	1273	1273	−1273	1273	1272

Note: (1) Standard errors are in parentheses. (2) * *p* < 0.1; ** *p* < 0.05; *** *p* < 0.01. (3) The result is estimated by fixed effect model with panel data. (4) We also control marriage, household number, log total expenditure and individual fixed effect.

**Table A8:** Effect of retirement on SAH after controlling channel variable.

	CV Activity	CV Activity	CW More 10 min	CW More 10 min
	SAH (Before)	SAH (After)	SAH (Before)	SAH (After)
	(1)	(2)	(3)	(4)
Retirement	0.515 *	0.472 *	0.515 *	0.477 *
	−0.279	−0.271	−0.279	−0.281
Vigorous activity		−0.146		
		−0.097		
Walking more 10 min				0.156 **
				−0.07
Marriage	0.331 *	0.309 *	0.331 *	0.341 **
	−0.176	−0.164	−0.176	−0.171
House hold num	0.039	0.039	0.039	0.037
	−0.031	−0.031	−0.031	−0.03
Log total expenditure	−0.021	−0.021	−0.021	−0.031
	−0.036	−0.035	−0.036	−0.035
Age	0.060 ***	0.061 ***	0.060 ***	0.050 ***
	−0.015	−0.015	−0.015	−0.015
Age2/100	0.215	0.152	0.215	0.21
	−0.338	−0.333	−0.338	−0.329
Cons	0.18	0.242	0.18	0.154
	−0.335	−0.332	−0.335	−0.326
N	1273	1273	1273	1273

Note: (1) Standard errors are in parentheses. (2) * *p* < 0.1; ** *p* < 0.05; *** *p* < 0.01. (3) The result is estimated by fixed effect model with panel data. (4) We also control marriage, household number, log total expenditure and individual fixed effect. (5) CV activity: Controlling Vigorous activity; CW more 10 min: Controlling Walking more 10 min.
